# Pre‐Percutaneous Coronary Intervention C‐Reactive Protein Levels and In‐Stent Restenosis: A Systematic Review and Meta‐Analysis

**DOI:** 10.1002/hsr2.70757

**Published:** 2025-04-29

**Authors:** Himanshu Rai, Renitha Reddi, J. J. Coughlan, Rory Durand, Daniel O'Callaghan, Roisin Colleran, Robert A. Byrne

**Affiliations:** ^1^ Department of Cardiology and Cardiovascular Research Institute (CVRI) Dublin Mater Private Network Dublin Ireland; ^2^ School of Pharmacy and Biomolecular Sciences RCSI University of Medicine and Health Sciences Dublin Ireland; ^3^ School of Medicine RCSI University of Medicine and Health Sciences Dublin Ireland

**Keywords:** bare metal stent, C‐reactive protein, drug eluting stent, in‐stent restenosis, percutaneous coronary intervention

## Abstract

**Introduction:**

C‐reactive protein (CRP) is an inflammatory biomarker, implicated in the pathogenesis of atherosclerotic lesion formation, plaque rupture, and coronary thrombosis. The relationship between preprocedural CRP levels and subsequent development of in‐stent restenosis (ISR) after percutaneous coronary intervention (PCI) however remains uncertain. Against this background, we performed a systematic review and meta‐analysis, investigating the association between baseline CRP levels and the incidence of ISR after PCI.

**Methods:**

Relevant published studies were identified following a PubMed, EMBASE, MEDLINE, Scopus and Web of Knowledge databases search until April 30, 2024. To be included, studies had to be original research with: (i) angiographically determined ISR group, (ii) angiographically or clinically determined no‐ISR group, and (iii) CRP levels measured before the index PCI procedure for both groups. The mean difference in baseline CRP levels and associated 95% confidence interval (CI) between the ISR and no‐ISR groups was ascertained for each included study. The pooled standard mean difference (SMD) and its 95% CI was derived after pooling study‐level results using a random effects model, employing a *Z*‐test. Begg's funnel plots and Egger's test were used for publication bias assessment.

**Results:**

Out of a total of 1018 unique results, 19 studies, with a total sample size of 4744 patients (1154 restenosis cases/3590 controls), were included for quantitative synthesis. ISR group had higher baseline CRP levels (SMD = 0.41, 95% CI = 0.16, 0.66 mg/L, *p* = 0.001) in comparison to the no‐ISR group. No evidence of publication bias was detected either visually by Begg's funnel plots or by Egger's test (*p* = 0.08). Our leave‐one‐out sensitivity analysis further attested our obtained associations.

**Conclusions:**

The present systematic review and meta‐analysis suggests a significant association of elevated pre‐PCI CRP with subsequent angiographically confirmed ISR. These results warrant further validation in dedicated large cohorts, ideally in a prospective setting.

## 1 Introduction

Coronary in‐stent restenosis (ISR) constitutes as an indication for 5%–10% of all percutaneous coronary intervention (PCI) procedures in contemporary general clinical practice, even after progressive evolutions in stent designs, associated PCI tools and techniques as well as advances in secondary prevention treatment strategies [1, 2]. Despite considerable reductions in the short‐to‐medium term incidence of ISR over the years, the absolute number of cases presenting with ISR represents a significant challenge for healthcare apparatus around the world [3]. ISR is traditionally defined as a reduction of luminal diameter of ≥ 50% within the stented segment or within 5 mm of proximal and distal vessel segments, on either side of the the stent [4]. The incidence of ISR is assessed visually in the catheterization laboratory or more precisely using offline quantitative coronary angiography (QCA), which is considered as the current gold standard [4]. Use of novel adjunct intravascular imaging techniques including optical coherence tomography and intravascular ultrasound are also finding traction in modern clinical practice for detection, sizing, and PCI optimization of ISR lesions.

Inflammation plays a central role in pathogenesis of ISR [5], and C‐reactive protein (CRP) is a potent marker of inflammation. CRP is an acute phase protein, and both the Centers for Disease Control and Prevention (CDC) and American Heart Association (AHA) have indicated that both average‐risk (1–3 mg/L), and high‐risk (> 3 mg/L) serum high‐sensitivity CRP (hs‐CRP) levels [6] are associated with significantly higher risk of cardiovascular death, myocardial infarction (MI), or stroke in stable patients with stable coronary artery disease, irrespective of coronary intervention, baseline characteristics or treatment arm, in a randomized setting [7]. Elevated baseline CRP levels has been found to be associated with increased risk of stent thrombosis, death, and MI in a large prospective study of 2691 PCI patients receiving drug eluting stents [8], while higher baseline hs‐CRP levels in patients undergoing PCI with sirolimus eluting stents have been shown to predict the 10‐ year composite endpoint of death or MI infarction in an all‐comers registry [9].

The relationship between baseline CRP levels with the incidence of ISR remains contentious. There have been five meta‐analyses published on this subject, amongst which three affirmed an association [10, 11, 12], whilst two did not [13, 14]. Against this background, we performed an updated study‐level systematic review and meta‐analysis, investigating the association between baseline CRP levels and the incidence of ISR after PCI.

## 2 Materials and Methods

The present meta‐analysis was conducted in accordance with the PRISMA statement (Preferred Reporting Items for Systematic Reviews and Meta‐Analyses) [15], as well as the HuGE Review Handbook, version 1.0 [16].

### 2.1 Search Strategy

A systematic search of online databases of the US National Institutes of Health (PubMed), EMBASE, MEDLINE, Scopus, Cochrane library and Web of Science was undertaken in order to identify relevant articles published online until April 30, 2024. Specific MESH headings in combination with open text fields were used to create online search strings for the databases listed above. Only original articles involving human subjects, with the results published in English language were considered for inclusion. Specific search headings used to build the search strings for different databases were “C‐reactive protein” OR “CRP” OR “high sensitivity C‐Reactive protein” OR “hs‐CRP” paired with “coronary restenosis” OR “restenosis” OR “coronary in‐stent restenosis” OR “coronary ISR” OR “ISR”. Reference lists of included papers and published meta‐analyses on the subject were also reviewed to identify additional articles for potential inclusion.

Study selection was performed using a hierarchical assessment model, initially starting with assessment of study titles for relevance, followed by abstracts, and subsequently full texts.

### 2.2 Inclusion and Exclusion Criteria

Qualifying studies had to have a clearly defined ISR (case) and no‐ISR (control) group, where the definition of ISR was based either on visual estimation by operators or using offline quantitative coronary angiography (QCA), with ≥ 50% stenosis inside the previously stented segment. The incidence of target lesion revascularization was considered as a surrogate for ISR. Conversely, no‐ISR controls were defined as cases with < 50% stenosis visually/via QCA or no incidence of TLR during post‐stenting follow‐up. No restrictions were applied on the design of the included studies. No restrictions were put either on: (i) the assay type (CRP or hs‐CRP), or (ii) the duration of the relook angiogram after the index stenting, or (iii) the stent type implanted at time of index PCI—which could have been exclusively either—or a combination of drug eluting and/or bare metal stents.

### 2.3 Quality Assessment

Data skewness and quality assessment of the included studies was assessed using already published assessment scales and statistical methods.

#### 2.3.1 Data Skewness

Studies reporting pre‐PCI CRP levels as mean ± SD were automatically assumed to be originating from a non‐skewed data set. Data skewness was suspected in studies reporting data as median (1st quartile–3rd quartile), or median (minimum–maximum), and were tested using previously published statistical methods [17]. Identified studies with possibly skewed data sets were subsequently excluded from the present meta‐analysis, whereas studies detected with non‐skewed data sets were included after converting CRP the data into mean [18] and SD [19], using previously published statistical methods.

#### 2.3.2 Newcastle–Ottawa Scale

The Newcastle–Ottawa scale (NOS) (http://www.ohri.ca/programs/clinical_epidemiology/oxford.asp) was used for quality assessment of the included studies. NOS is a star‐based rating system, where a study can earn a maximum of 9 stars [20]. The NOS rating tool involves evaluation of studies in three specific domains, which relates to (a) selection methods of study participants, (b) comparability amongst cases and control groups, and (c) exposure and outcome. A study which earns 5–9 stars is assumed to be a good quality study, while a score of 0–4 stars is indicative of a poor‐quality study. Included studies were independently assessed using NOS by two authors and disagreements were later resolved by consensus.

### 2.4 Statistical Analysis

All statistical calculations were carried out using windows based RevMan version 5.4.1 (The Cochrane Collaboration, 2020) and GraphPad Prism for windows version 10.1.2 (GraphPad software LLC).

#### 2.4.1 Summary Effect Measures

Pooled standard mean difference (SMD) with its 95% CI (in mg/L) was the chosen summary effect measure to test the association of baseline CRP levels with the incidence of ISR. SMD (and its 95% CI) were estimated for each study after which a *Z*‐test was employed to obtain a pooled SMD (95% CI), using bivariate, random effects model (DerSimonian–Laird method) [21]. A pooled *p*‐value of < 0.05 indicated statistical significance and the corresponding *Z*‐value indicated the level of association.

#### 2.4.2 Heterogeneity Assessment

*Q*‐test was performed for heterogeneity assessment, where the resultant Higgins *I* ^2^ statistics (*I* ^2^) and Cochrane's Q statistics (*P*_Q_) were used as indicators of in‐group heterogeneity. A resultant *P*_Q_ of < 0.01 indicated significant heterogeneity, while *I* ^2^ values of 25%, 50%, and 75% indicated low, moderate, and high heterogeneity respectively [22]. Summary effect measures in heterogenous groups, yielding a *P*_Q_ ≤ 0.01 as well as *I* ^2^ ≥ 50%, were derived using random effect model. Conversely, fixed effect model for analysis was used where there was no/low evidence of heterogeneity [23].

#### 2.4.3 Publication Bias Assessment

Visual assessment of publication bias amongst the studied groups was done using Begg's funnel plot [24], while statistical estimates of the same were derived using Egger's test [25]. Each point in the funnel plot represents the estimated OR for each study plotted against its standard error (SE). These plots appear largely symmetrical around the central line representing pooled OR (obtained using random effects), in groups with limited/no publication bias. On the other hand, pronounced plot asymmetry indicated existence of substantial bias. Statistical estimates for the existence of publication bias were derived using Egger's test, where a resultant *p*‐value of < 0.05 indicated significant publication bias.

#### 2.4.4 Sensitivity Analysis

Leave‐one‐out analysis was performed, where the analysis was repeated after omitting one study after another, to test for significant alterations in the pooled ORs or its 95% CI from the original estimates.

## 3 Results

**Figure 1:**
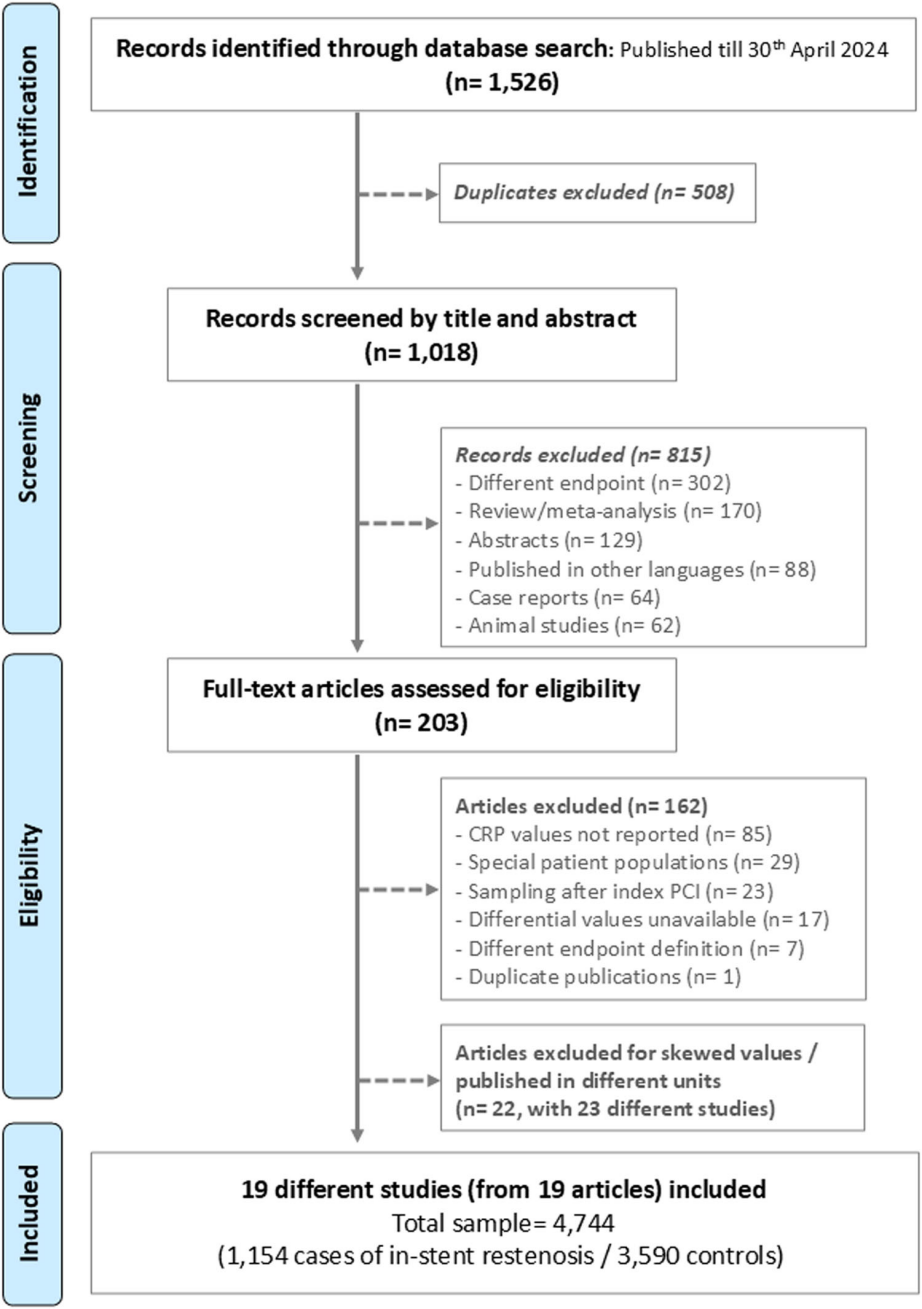
PRISMA study flow. CRP, C‐Reactive protein; PCI, percutaneous coronary intervention.

Amongst a total of 1018 unique articles screened, full texts of 203 articles were assessed for eligibility. Amongst these, 162 articles were excluded for a variety of reasons, listed in the PRISMA flow diagram (Figure 1), while a further 22 articles (with 23 different studies) were excluded for skewed CRP values/CRP values published in different units than mg/L or equivalent. (Table S1). The remaining 19 articles, reporting as many studies, with a total sample size of 4744 patients (1154 ISR cases and 3590 patient controls), were included for quantitative synthesis [1, 26, 27, 28, 29, 30, 31, 32, 33, 34, 35, 36, 37, 38, 39, 40, 41, 42, 43].

Table 1 lists the baseline characteristics of the included studies, such as sample size, type and duration of follow‐up angiogram from index stenting, type of CRP assay, data units for reporting CRP results as well as NOS rating for individual studies. All included studies were adjudged to be good quality, receiving NOS rating ranging from 5–9 stars.

**Table 1:** Baseline characteristics of included studies.

Name	Year	Sample size (ISR/no‐ISR)	Follow‐up angiogram	Duration between index stenting and follow‐up angiogram	Assay type	Data expressed as (units)	NOS rating
Segev et al. [26]	2004	32/184	Planned	6 months	hs‐CRP	mean ± SD (mg/L)	8/9
Yip et al. [27]	2005	22/55	Planned	6 months	hs‐CRP	mean ± SD (mg/L)	8/9
Turker et al. [28]	2006	10/26	Planned	6 months	hs‐CRP	mean ± SD (mg/L)	7/9
Khosravi et al. [29]	2009	46/82	Planned	6 months	CRP	mean ± SD (mg/L)	8/9
Ari et al. [30]	2010	27/65	Planned	6 months	hs‐CRP	mean ± SD (mg/L)	8/9
Park et al. [31]	2011	35/35	Clinically driven	6–9 months	CRP	mean ± SD (mg/L)	6/9
Hage et al. [32]	2011	35/47	Planned	6–8 months	hs‐CRP	median, 1st and 3rd quartile (mg/L)	9/9
Yildiz et al. [33]	2014	131/138	Clinically driven	within 24 months	CRP	mean ± SD (mg/L)	8/9
Zhao et al. [34]	2015	45/247	Clinically driven	6–9 months	CRP	mean ± SD (mg/L)	6/9
Li et al. (a) [35]	2015	35/65	Planned	8 months	hs‐CRP	mean ± SD (mg/L)	8/9
Tanindi et al. [36]	2015	32/283	Planned	6–12 months	CRP	mean ± SD (µg/ml, equals mg/L)	6/9
Pantovic et al. [1]	2015	8/36	Planned	6 months	hs‐CRP	mean ± SD (mg/L)	7/9
Lee et al. [37]	2017	162/910	Clinically driven	within 12 months	hs‐CRP	mean ± SD (mg/L)	6/9
Kurtul [38]	2018	166/192	Clinically driven	median 24 months (range 6–60 months)	hs‐CRP	median, 1st and 3rd quartile (mg/L)	6/9
Qian et al. [39]	2018	118/143	Clinically driven	within 12 months	hs‐CRP	mean ± SD (mg/L)	5/9
Xu et al. [40]	2019	95/517	Clinically driven	6–12 months	hs‐CRP	mean ± SD (mg/L)	5/9
Aksu et al. [41]	2019	45/45	Clinically driven	median 44 months	CRP	median, 1st and 3rd quartile (mg/L)	7/9
Li et al. (b) [42]	2019	72/344	Clinically driven	14.4 ± 3.3 months	hs‐CRP	mean ± SD (mg/L)	5/9
Song et al. [43]	2021	38/176	Planned	24 months	hs‐CRP	median, 1st and 3rd quartile (mg/L)	9/9

Abbreviations: CRP, C‐reactive protein; DES, drug eluting stent; hs‐CRP, high‐sensitivity C‐reactive protein; ISR, In‐stent restenosis; NOS, Newcastle–Ottawa scale; SD, standard deviation.

**Figure 2:**
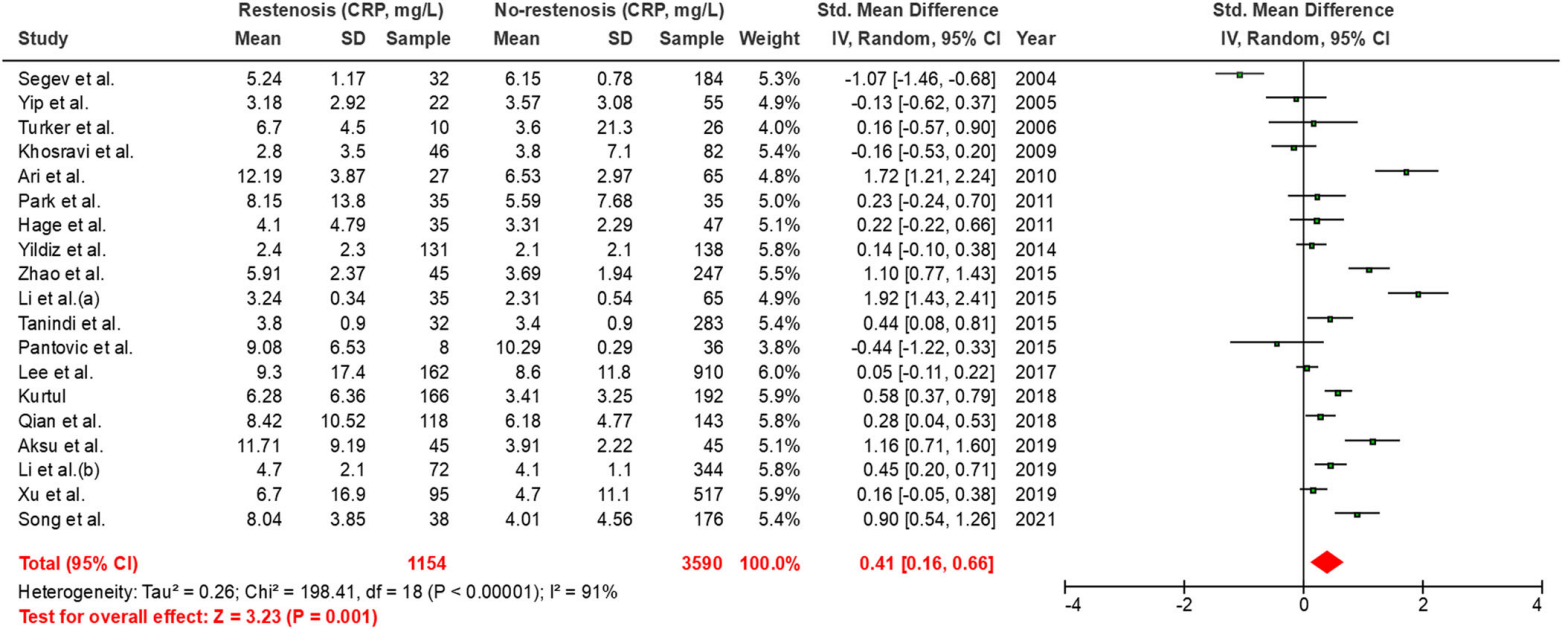
Forest plot displaying the main results. CRP, C‐reactive protein; ISR, in‐stent restenosis.

**Figure 3:**
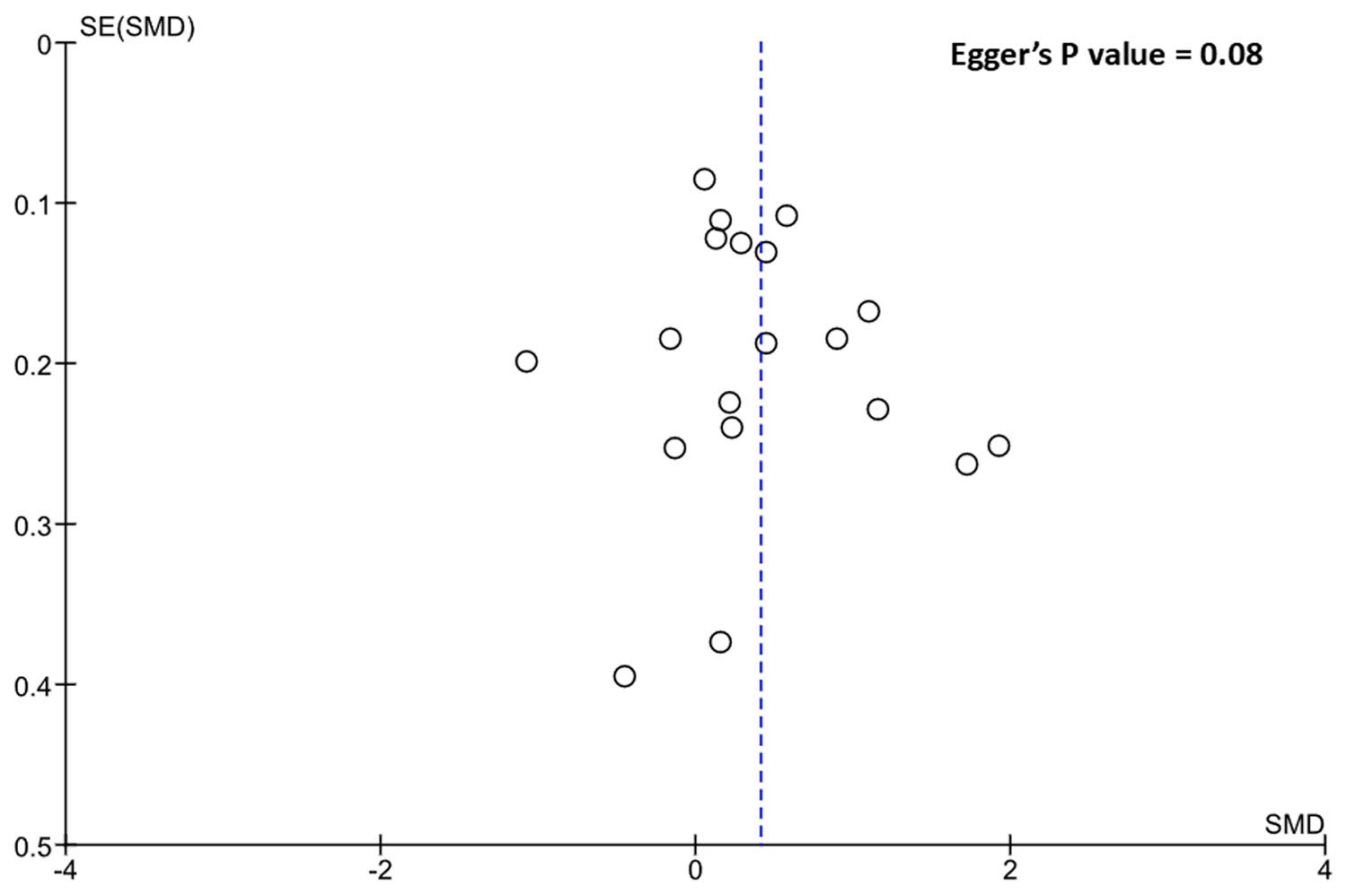
Publication bias assessment results.

**Figure 4:**
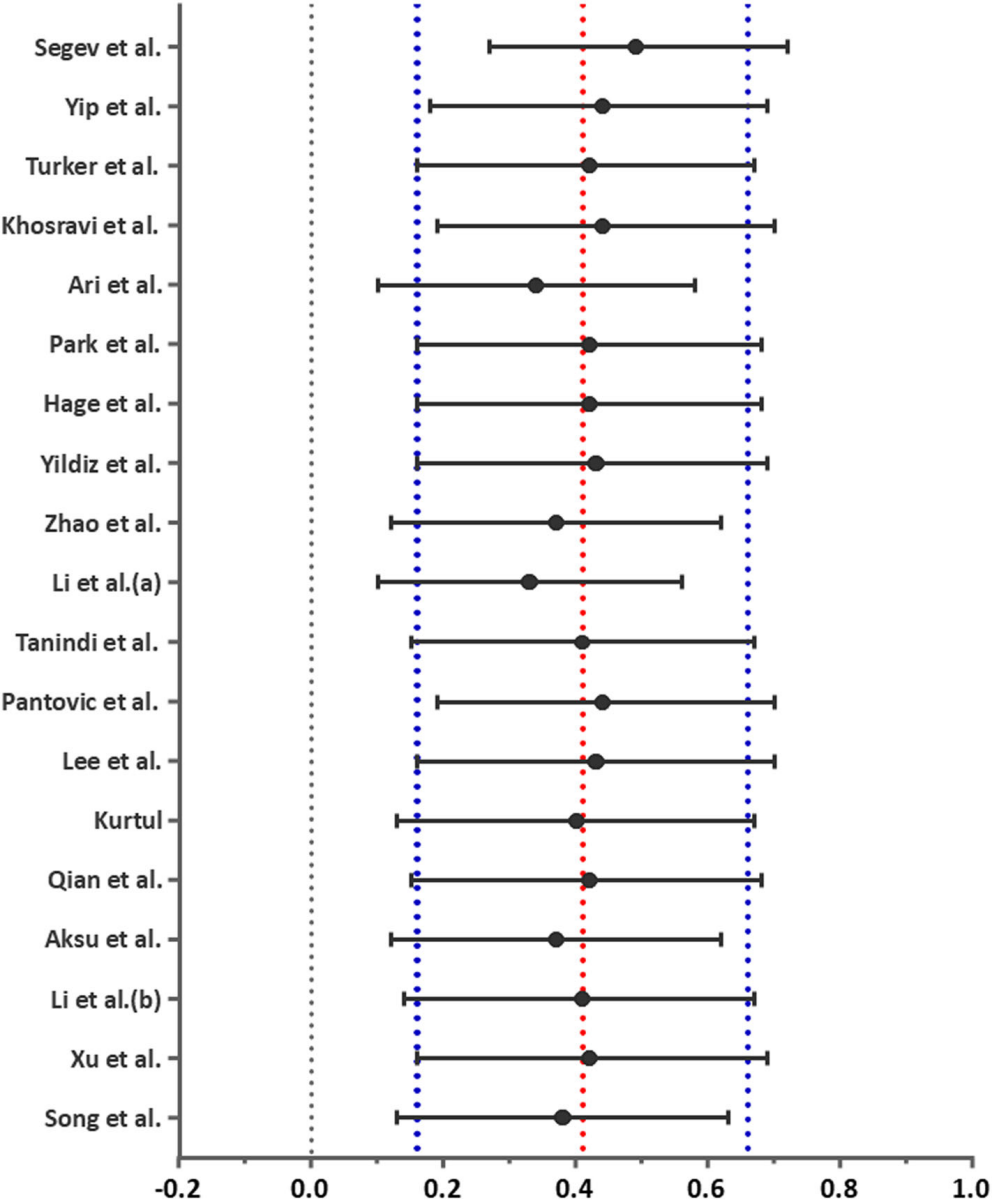
Graph displaying sensitivity analysis results.

Our pooled analysis which included 19 studies [1, 26, 27, 28, 29, 30, 31, 32, 33, 34, 35, 36, 37, 38, 39, 40, 41, 42, 43], and performed using random effects, demonstrated a significant association between higher baseline CRP levels and the incidence of angiographically confirmed coronary restenosis (SMD = 0.41; 95% CI = 0.16, 0.66; *p* = 0.001. (Figure 2). No evidence of publication bias was seen, visually using Begg's funnel plots or using Egger's test (*p* = 0.08) (Figure 3). Our obtained results remained consistent in our leave‐one‐out sensitivity analysis (Figure 4).

## 4 Discussion

Our present meta‐analysis of 19 eligible studies involving a sample of 1154 ISR cases and 3590 controls confirmed the association between higher baseline CRP values and the incidence of angiographically confirmed ISR after PCI. No evidence of publication bias was detected. The robustness of our obtained results was further confirmed by our sensitivity analysis.

Inflammation plays a key role in atherosclerotic plaque formation [44] and has been associated with an increased risk of future atherothrombotic events [45] and even restenosis [46] after PCI. CRP, an acute‐phase protein, produced primarily by hepatocytes [47], is perhaps the most studied circulating inflammatory biomarker in relation to atherosclerotic cardiovascular disease. The role of CRP in ISR has also been investigated by several prospective or retrospective, randomized, case‐control, and cohort studies, with the extracted data pooled and post‐synthesized by at least five published study‐level meta‐analyses to date [10, 11, 12, 13, 14]. Discrepancies in the results from these published meta‐analyses [10, 11, 12, 13, 14], motivated this up to date meta‐analysis. It is noteworthy that the two latest meta‐analyses on the subject only included six [10] and nine [14] studies respectively, reporting diametrically opposite results. In contrast, our present updated analysis included a total of 19 studies and produced robust results, validated by a sensitivity analysis and without any evidence of publication bias.

### 4.1 Triggering of the Inflammation Cascade and CRP Production

Several biochemical changes occur in the human body during highly inflammatory states (broadly referred to as acute phase response) where there are changes in the concentrations of several plasma proteins, including CRP. However, it is still debated whether CRP has a direct causal relationship with progression of atherosclerosis [48, 49, 50, 51, 52], or if the increase in plasma CRP is a response to the inflammatory processes involved in atherosclerotic CAD [53, 54, 55]. Several single‐nucleotide polymorphisms residing within the CRP gene located at 1q23.2, in the long arm of chromosome 1, has been shown to genetically determine a marked increase in CRP levels [56]. Factors such as age, gender, smoking, body weight, lipid levels, and blood pressure also have been known to alter plasma CRP levels [57]. In addition, triggering of the inflammatory cascade following the activation of NLRP3 (Nod‐Like‐Receptor‐Protein‐3) inflammasome, in response to chronic inflammatory conditions such as diabetes and atherosclerosis also has been known to up‐regulate plasma CRP levels [58], via triggering a central cascade of inflammatory signaling represented by interleukin (IL)‐1 β, IL‐6, and CRP [59, 60]. Activation of NLRP3 inflammasome, a vital component of the innate immune system, activates the cysteine protease caspase‐1, which proteolyzes three proteins: pro‐IL‐1 β, pro‐IL‐18, and gasdermin D [58]. Pro‐IL‐1β and pro‐IL‐18 are then converted into active forms of pro‐inflammatory IL‐1β and IL‐19, respectively. Pro‐inflammatory IL‐1β produced in the myeloid cells incudes IL‐6 synthesis [61, 62], both of which in turn stimulate production of CRP in the hepatocytes, which circulates as a pentamer (pCRP) in plasma [47]. CRP is essentially a pentraxin, first described by Tillet and Francis in the year 1930 [63], consists of five identical, non‐covalently associated subunits, arranged symmetrically around a central pore [64]. Monomeric structural isoforms of CRP (mCRP) seem to be involved in most pro‐inflammatory actions, and are produced following natural dissociation from its native pentameric form (pCRP) [65].

### 4.2 Baseline CRP Levels and Post‐PCI Prognosis

In line with the results of our present study, strong relationships between higher baseline CRP levels and worse prognosis after index PCI have been reported, including in the incidence of all‐cause death as well as MI in a retrospective cohort of 7026 patients [45], and a composite primary endpoint of death, MI and stroke in a retrospective review of a prospective, randomized EXCEL trial involving 999 patients [66]. Elevated baseline CRP levels have also been found to be predictive factor of a worse short‐term outcome after PCI, including 30‐day death or myocardial infarction [67] as well as short and long‐term mortality after non ST acute coronary syndrome treated with early revascularization in 1042 consecutively stented cases [68]. The molecular basis of such events and involved inflammatory processes in stented atherosclerotic lesions have yet to be elucidated. In a study population of 276 cases after coronary stenting which all received 6‐month check angiogram for restenosis assessment, logistic regression analysis suggested significant associations of incidence of stent restenosis, with second and third tertiles (individually and combined) as compared to the first tertile of preprocedural CRP levels [69]. While other post PCI MACE events such as MI and deaths, can at least in part be attributed to untreated coronary lesions, the association of baseline CRP levels with angiographically confirmed ISR in the present study hints towards a possible mechanistic processes. Suggested pro‐inflammatory properties of CRP includes: (i) activation of the complement cascade [70, 71], (ii) induction in monocytes of inflammatory cytokines and tissue factor [70, 71], as well as (iii) shedding of the IL‐6 receptor [72]. The CRP elevation response to tissue injury may worsen tissue damage in certain settings [73], also perhaps after coronary stenting, which in part suggests its association with increased incidence of ISR in cases carrying residual inflammation indicated by higher baseline CRP levels.

### 4.3 Available Treatment Options

Improvement in PCI outcomes including reductions in post‐PCI incidence of major adverse cardiovascular events (including ISRs requiring TLRs), amongst patients with high‐risk baseline CRP levels, defined as ≥ 3 mg/L as per the 2003 CDC/AHA statement [6], could in principle be achieved using several pharmacological interventions. These include statins alone or with adjunct lipid‐lowering therapy, as well as direct targeting of NLRP3 inflammasome and upstream links residing in the IL1‐β/IL‐6/CRP axis, as well as directly by anti‐CRP compounds, which albeit, are still under development.

Numerous randomized clinical trials (RCTs) including PROVE IT‐TIMI 22 [74], IMPROVE‐IT [75], REVERSAL [76], JUPITER [77], FOURIER [78] etc., have reported a marked reduction in subclinical inflammation through CRP lowering, as one of the pleotropic effects of statins (alone or in combination with other lipid lowering agents), at least in some proportion of treated patients, clinically demonstrated by up to 28%–33% reduction in follow‐up major adverse cardiovascular events (MACE) [74, 75]. Colchicine, a NLRP3 inflammasome inhibitor has been tested in several RCTs including COLCOT [79], and LoDoCo2 [80], both of which reported significant reduction in MACE rates [79, 80], along with concomitant reductions in CRP levels [79]. Primary results of COLCRP, a prospective, placebo‐controlled RCT exploring the effect of three different doses of colchicine on hs‐CRP levels on post‐PCI patients are yet to be published, but promises more insights into targeted hs‐CRP lowering as well as dose effects of colchicine (NCT06078904). Canakinumab, an IL1‐β antagonist tested in CANTOS, a placebo controlled RCT, also has shown great promise and reported significant reductions in MACE rates as well as hs‐CRP levels amongst patients with a history of MI and baseline hs‐CRP levels ≥ 2 mg/L, irrespective of their revascularization status [81]. Recently, ziltivikimab, an IL‐6 inhibiting monoclonal antibody, has been tested in the RESCUE trial amongst participants with moderate to severe chronic kidney disease and an hs‐CRP levels of > 2.0 mg/L, where treatment for 12 weeks resulted in 77%–92% reduction in the median CRP levels [82]. Potential of this IL‐6 inhibitor in reduction of CRP and MACE rates amongst patients with cardiovascular disease, chronic kidney disease and baseline hs‐CRP levels of > 2.0 mg/L, is currently being tested in the ZEUS trial; results of which are highly anticipated [83]. There are some anti‐CRP compounds, which are mostly under development and being tested in animal models [84, 85, 86, 87, 88, 89] or phase II clinical studies amongst humans [85, 90, 91, 92], and are yet to be commercially available. The effectiveness of these in improving post‐PCI prognosis in clinical setting is yet to be ascertained.

### 4.4 Limitations

The present study‐level meta‐analysis has several limitations that warrant listing. First, our included study group consisted of non‐randomized, retrospective/cohort studies with relatively small–moderate sample size. Historically, non‐randomized study designs are known to produce biased results, due to unadjusted confounders. In addition, bias in results could have also been introduced due to “small‐study effects”—a phenomenon in which studies with small sample size show different, often larger, treatment effects as compared to large ones. Second, included studies included patients with a variety of PCI indications, including acute coronary syndromes, especially acute MIs, which is perhaps one of the most important sources of heterogeneity in CRP levels. Third, most included studies visually assessed the presence of ISR, and there was no centralized core lab for restenosis assessment, which could have contributed towards selection bias. Fourth, included studies implanted a variety of stents including BMS which classically are more prone to ISR than the contemporary generation DES. Fifth, variations in duration between index PCI and relook angiograms would also have contributed towards heterogeneity. Sixth, the extent of pleotropic effects of widely prevalent secondary prevention strategies which includes statin treatment on CRP levels are also unknown and are also unaccounted for. Seventh, effects of other risk factors for ISR like diabetes mellitus, lesion complexity, and evolution of stents and stent materials, with constantly evolving smaller strut sizes and variety of drugs and coating platforms in different studies over the years were also unaccounted for. All aforementioned factors, including underlying conditions such as cancers, active bacterial/viral infections, connective tissue diseases, ongoing hormonal or chemotherapies which would have affected baseline CRP levels, were unaccounted for and would have contributed to heterogeneity amongst our study group, and could have introduced/contributed towards bias in our produced results.

## 5 Conclusions

The present systematic review and study‐level meta‐analysis of non‐randomized studies demonstrates an association between higher baseline CRP values and the subsequent incidence of angiographically confirmed ISR after PCI. Whether treatment for elevated CRP at baseline reduces subsequent risk of ISR, may warrant further investigation.

## Author Contributions

**Himanshu Rai:** conceptualization, data curation, formal analysis, methodology, project administration, software, supervision, validation, visualization, writing – original draft, writing – review and editing. **Renitha Reddi:** data curation and resources. **J.J. Coughlan:** supervision, visualization, writing – original draft, writing – review and editing. **Rory Durand:** writing – review and editing. **Daniel O'Callaghan:** writing – review and editing. **Roisin Colleran:** writing – review and editing. **Robert A. Byrne:** methodology, supervision, validation, writing – review and editing.

## Conflicts of Interest

Professor Robert A. Byrne reports research funding to the institution from Abbott Vascular, Biosensors, Boston Scientific, and Translumina. All other authors declare no conflicts of interest.

## Transparency Statement

The lead author Himanshu Rai affirms that this manuscript is an honest, accurate, and transparent account of the study being reported; that no important aspects of the study have been omitted; and that any discrepancies from the study as planned (and, if relevant, registered) have been explained.

## Supporting information

Supplementary Table 1. List of excluded articles for skewed values/published in different units.

## Data Availability

The authors confirm that the data supporting the findings of this study are available within the article [and/or] its Supporting Information S1.
