# Supplementary material for: Pre‐Percutaneous Coronary Intervention C‐Reactive Protein Levels and In‐Stent Restenosis: A Systematic Review and Meta‐Analysis
Source: Health Sci Rep. 2025 Apr 29;8(5):e70757. doi: 10.1002/hsr2.70757 (PMC12040731; doi:10.1002/hsr2.70757)
Supplement: Supplementary file 1 — Supplementary Table 1. List of excluded articles for skewed values/published in different units. [file HSR2-8-e70757-s001.docx]

**Supplementary Table 1. List of excluded articles for skewed values / published in different units**

| **Name** | **Year** | **Sample size (ISR/no-ISR)** | **Data expressed as** | **Units** | **Reason for exclusion** |
| --- | --- | --- | --- | --- | --- |
| Gomma *et al.*[1] | 2004 | 45/88 | median, 1^st^ and 3^rd^ quartile | mg/L | Skewed data |
| Skowasch *et al.*[2] | 2005 | 61/158 | median, Interquartile range | mg/L | Skewed data |
| Kaehler *et al.*[3] | 2005 | 57/259 | mean, standard error of mean | mg/L | Skewed data |
| Karaca *et al.*[4] | 2005 | 31/43 | median, Interquartile range, minimum and maximum | mg/L | Skewed data |
| Yan *et al.*[5] | 2006 | 38/112 | mean±SD | ng/ml | Different units |
| Ulus *et al.*[6] | 2007 | 60/60 | median, 1^st^ and 3^rd^ quartile | mg/L | Skewed data |
| Saleh and Tornvall[7] | 2007 | 139/711 | median, 1^st^ and 3^rd^ quartile | mg/L | Skewed data |
| Yan *et al.*[8] | 2007 | 29/91 | mean±SD | ng/ml | Different units |
| Ziakas *et al.*[9] | 2009 | 7/33 | mean±SD | mg/dl | Different units |
| Xu *et al.*(a)[10] | 2011 | 48/255 | median, 1^st^ and 3^rd^ quartile | mg/L | Skewed data |
| Xu *et al.*(b)[11] | 2012 | 94/307 | median, interquartile range | mg/L | Skewed data |
| Katsaros *et al.*[12] | 2014 | 12/73 | median, 1^st^ and 3^rd^ quartile | mg/L | Skewed data |
| Ucar *et al.*[13] | 2016 | 225/243 | mean±SD | mg/dl | Different units |
| Qin *et al.*[14] | 2017 | 132/1074 | median, 1^st^ and 3^rd^ quartile | mg/L | Skewed data |
| Murat *et al.*[15] | 2017 | 117/156 | median, 1^st^ and 3^rd^ quartile | mg/L | Skewed data |
| Seo *et al.*[16] | 2018 | 27/214 | mean±SD | mg/dl | Different units |
| Rencuzogullari *et al.*[17] | 2019 | 110/338 | median, 1^st^ and 3^rd^ quartile | mg/dl | Different units |
| Wu *et al.*[18] | 2019 | 45/169 | median, 1^st^ and 3^rd^ quartile | mg/L | Skewed data |
| Zheng *et al.*(a)[19] | 2019 | 40/354 | mean±SD | mg/dl | Different units |
| Zheng *et al.*(b)[19] | 2019 | 19/335 | mean±SD | mg/dl | Different units |
| Zhao *et al.*[20] | 2020 | 37/361 | median, 1^st^ and 3^rd^ quartile | mg/L | Skewed data |
| Feng *et al.*[21] | 2022 | 20/215 | median, 1^st^ and 3^rd^ quartile | mg/L | Skewed data |
| Osken et al.[22] | 2024 | 264 / 639 | mean±SD | mg/dl | Different units |

Abbreviations: ISR, in-stent restenosis, mean±SD, mean ± standard deviation.

**REFERENCES**

1. Gomma AH, Hirschfield GM, Gallimore JR, Jr., Lowe GD, Pepys MB, Fox KM. Preprocedural inflammatory markers do not predict restenosis after successful coronary stenting. Am Heart J. 2004;147(6):1071-7. Epub 2004/06/17. doi: 10.1016/j.ahj.2003.10.050. PubMed PMID: 15199358.

2. Skowasch D, Jabs A, Andrie R, Luderitz B, Bauriedel G. Progression of native coronary plaques and in-stent restenosis are associated and predicted by increased pre-procedural C reactive protein. Heart (British Cardiac Society). 2005;91(4):535-6. Epub 2005/03/18. doi: 10.1136/hrt.2004.037317. PubMed PMID: 15772225; PubMed Central PMCID: PMCPMC1768800.

3. Kaehler J, Haar A, Schaps KP, Gaede A, Carstensen M, Schalwat I, et al. A randomized trial in patients undergoing percutaneous coronary angioplasty: roxithromycin does not reduce clinical restenosis but angioplasty increases antibody concentrations against Chlamydia pneumoniae. Am Heart J. 2005;150(5):987-93. Epub 2005/11/18. doi: 10.1016/j.ahj.2005.01.024. PubMed PMID: 16290983.

4. Karaca I, Aydin K, Yavuzkir M, Ilkay E, Akbulut M, Isik A, et al. Predictive value of C-reactive protein in patients with unstable angina pectoris undergoing coronary artery stent implantation. The Journal of international medical research. 2005;33(4):389-96. Epub 2005/08/18. doi: 10.1177/147323000503300404. PubMed PMID: 16104442.

5. Yan JC, Ma GS, Zhu J, Feng Y, Luo D, Wu ZG, et al. The clinical implications of increased coexpression of CD40-CD40 ligand system and C-reactive protein in patients after percutaneous coronary intervention. Clinica chimica acta; international journal of clinical chemistry. 2006;374(1-2):140-1. Epub 2006/07/06. doi: 10.1016/j.cca.2006.05.025. PubMed PMID: 16820144.

6. Ulus T, Yildirir A, Demirtas S, Demir O, Sade LE, Bozbas H, et al. Serum gamma-glutamyl transferase activity: a new marker for stent restenosis? Atherosclerosis. 2007;195(2):348-53. Epub 2006/11/08. doi: 10.1016/j.atherosclerosis.2006.09.025. PubMed PMID: 17087967.

7. Saleh N, Tornvall P. Serum C-reactive protein response to percutaneous coronary intervention in patients with unstable or stable angina pectoris is associated with the risk of clinical restenosis. Atherosclerosis. 2007;195(2):374-8. Epub 2006/11/28. doi: 10.1016/j.atherosclerosis.2006.10.026. PubMed PMID: 17126343.

8. Yan JC, Ding S, Liang Y, Ma GS, Zhu J, Feng Y, et al. Relationship between upregulation of CD40 system and restenosis in patients after percutaneous coronary intervention. Acta Pharmacol Sin. 2007;28(3):339-43. Epub 2007/02/17. doi: 10.1111/j.1745-7254.2007.00520.x. PubMed PMID: 17302995.

9. Ziakas A, Gavrilidis S, Giannoglou G, Souliou E, Koskinas K, Gemitzis K, et al. Kinetics and prognostic value of inflammatory-sensitive protein, IL-6, and white blood cell levels in patients undergoing coronary stent implantation. Medical science monitor : international medical journal of experimental and clinical research. 2009;15(4):CR177-84. Epub 2009/04/01. PubMed PMID: 19333202.

10. Xu YL, Li JJ, Xu B, Zhu CG, Yang YJ, Chen JL, et al. Role of plasma C-reactive protein in predicting in-stent restenosis in patients with stable angina after coronary stenting. Chin Med J (Engl). 2011;124(6):845-50. Epub 2011/04/27. PubMed PMID: 21518590.

11. Xu HY, Qiao SB, Zhang JF, Dong QT, Li JJ. Different impacts of C-reactive protein and lipid profile on coronary lesions following a percutaneous coronary intervention. Coron Artery Dis. 2012;23(3):181-7. Epub 2012/02/18. doi: 10.1097/MCA.0b013e3283519f44. PubMed PMID: 22336660.

12. Katsaros KM, Kastl SP, Krychtiuk KA, Hutter R, Zorn G, Maurer G, et al. An increase of VEGF plasma levels is associated with restenosis of drug-eluting stents. EuroIntervention. 2014;10(2):224-30. Epub 2013/10/31. doi: 10.4244/EIJV10I2A36. PubMed PMID: 24168783.

13. Ucar FM. A potential marker of bare metal stent restenosis: monocyte count - to- HDL cholesterol ratio. BMC Cardiovasc Disord. 2016;16(1):186. Epub 2016/10/08. doi: 10.1186/s12872-016-0367-3. PubMed PMID: 27716070; PubMed Central PMCID: PMCPMC5048646.

14. Qin Z, Zheng FW, Zeng C, Zhou K, Geng Y, Wang JL, et al. Elevated Levels of Very Low-density Lipoprotein Cholesterol Independently Associated with In-stent Restenosis in Diabetic Patients after Drug-eluting Stent Implantation. Chin Med J (Engl). 2017;130(19):2326-32. Epub 2017/08/25. doi: 10.4103/0366-6999.213575. PubMed PMID: 28836572; PubMed Central PMCID: PMCPMC5634084.

15. Murat SN, Yarlioglues M, Celik IE, Kurtul A, Duran M, Kilic A, et al. The Relationship Between Lymphocyte-to-Monocyte Ratio and Bare-Metal Stent In-Stent Restenosis in Patients With Stable Coronary Artery Disease. Clin Appl Thromb Hemost. 2017;23(3):235-40. Epub 2016/01/14. doi: 10.1177/1076029615627340. PubMed PMID: 26759373.

16. Seo DJ, Kim YK, Seo YH, Song IG, Kim KH, Kwon TG, et al. In-stent restenosis-prone coronary plaque composition: A retrospective virtual histology-intravascular ultrasound study. Cardiol J. 2018;25(1):7-13. Epub 2017/10/25. doi: 10.5603/CJ.a2017.0124. PubMed PMID: 29064537.

17. Rencuzogullari I, Karabag Y, Cagdas M, Karakoyun S, Seyis S, Gursoy MO, et al. Assessment of the relationship between preprocedural C-reactive protein/albumin ratio and stent restenosis in patients with ST-segment elevation myocardial infarction. Rev Port Cardiol (Engl Ed). 2019;38(4):269-77. Epub 2019/05/21. doi: 10.1016/j.repc.2018.08.008. PubMed PMID: 31104927.

18. Wu Y, Fu X. Comprehensive analysis of predictive factors for rapid angiographic stenotic progression and restenosis risk in coronary artery disease patients underwent percutaneous coronary intervention with drug-eluting stents implantation. Journal of clinical laboratory analysis. 2019;33(2):e22666. Epub 2018/09/18. doi: 10.1002/jcla.22666. PubMed PMID: 30221497; PubMed Central PMCID: PMCPMC6818547.

19. Zheng C, Kang J, Park KW, Han JK, Yang HM, Kang HJ, et al. The Predictors of Target Lesion Revascularization and Rate of In-Stent Restenosis in the Second-Generation Drug-Eluting Stent Era. J Interv Cardiol. 2019;2019:3270132. Epub 2019/11/28. doi: 10.1155/2019/3270132. PubMed PMID: 31772522; PubMed Central PMCID: PMCPMC6739790.

20. Zhao J, Wang X, Wang H, Zhao Y, Fu X. Occurrence and predictive factors of restenosis in coronary heart disease patients underwent sirolimus-eluting stent implantation. Ir J Med Sci. 2020;189(3):907-15. Epub 2020/01/29. doi: 10.1007/s11845-020-02176-9. PubMed PMID: 31989420.

21. Feng Q, Zhao Y, Wang H, Zhao J, Wang X, Shi J. A predictive model involving serum uric acid, C-reactive protein, diabetes, hypercholesteremia, multiple lesions for restenosis risk in everolimus-eluting stent-treated coronary heart disease patients. Front Cardiovasc Med. 2022;9:857922. Epub 2022/08/30. doi: 10.3389/fcvm.2022.857922. PubMed PMID: 36035940; PubMed Central PMCID: PMCPMC9403046.

22. Osken A, Polat F, Cakir B, Zengin A, Calik AN, Unal Dayi S, et al. Systemic immune inflammation index and its implication on in-stent restenosis among patients with acute coronary syndrome. Coron Artery Dis. 2024;35(3):209-14. Epub 2024/01/05. doi: 10.1097/MCA.0000000000001325. PubMed PMID: 38180335.
